# SET-CAN/NUP214 fusion gene in leukemia: general features and clinical advances

**DOI:** 10.3389/fonc.2023.1269531

**Published:** 2023-10-16

**Authors:** Jingyu Song, Huibo Li, Shengjin Fan

**Affiliations:** ^1^ Department of Hematology, The First Affiliated Hospital, Harbin Medical University, Harbin, China; ^2^ NHC Key Laboratory of Cell Transplantation, The First Affiliated Hospital, Harbin Medical University, Harbin, China

**Keywords:** SET-CAN/NUP214 fusion gene, leukemia, T-cell acute lymphoblastic leukemia (T-ALL), acute myeloid leukemia (AML), molecular anomaly, treatment, prognosis

## Abstract

*SET-CAN/NUP214* fusion is a recurrent event commonly observed in adult male patients diagnosed with T-cell acute lymphoblastic leukemia (T-ALL) and has occasionally been reported in other diseases such as acute myeloid leukemia (AML), myeloid sarcoma (MS), acute undifferentiated leukemia (AUL), chronic myeloid leukemia (CML) and B-cell acute lymphoblastic leukemia (B-ALL). This fusion gene is derived from chromosome del(9)(q34.11;q34.13) or t(9;9)(q34;q34) and may have an inhibitory effect on primitive progenitor differentiation. The prognosis of the reported patients is varied, with these patients often show resistance to chemotherapy regimens that include high doses of glucocorticoids. The optional treatment has not been determined, more cases need to be accumulated and evaluated. The scope of this review is to summarize the general features and prognostic significance in leukemia associated with the *SET-CAN/NUP214* fusion gene and to discuss the methods of detection and treatment, aiming at providing some useful references for relevant researchers in the field of blood tumor.

## 1 Introduction

Leukemia is a malignant clonal disease originating from hematopoietic stem and progenitor cells. Leukemia cells with proliferation and survival advantages proliferate and accumulate uncontrollably in the body, gradually replacing normal hematopoiesis and invading other organs and systems, resulting in a series of symptoms such as anemia, hemorrhage, infection and immersion. According to the degree of differentiation and maturation of leukemia cells and the natural course of disease, leukemia can be roughly divided into two categories: acute leukemia and chronic leukemia, and then divided into myelogenic/myeloid and lymphocytic/lymphoblastic according to the cell of origin.

*SET-CAN/NUP214* fusion gene is formed by del(9)(q34.11;q34.13) or t(9;9)(q34;q34) and has been identified in the LOUCY cell line of T -ALL and the MEGAL cell line of AML(1, 2). In 1992, Von Lindern et al. first identified the *SET-CAN/NUP214* fusion gene in a case of acute undifferentiated leukemia (AUL). Since then, with the development of detection technology and the deepening understanding of leukemia, subsequent cases of AML, MS, AUL, CML, and B-ALL have also been found (3–6). Overall, the disease experienced by most patients carrying *SET-CAN/NUP214* is T-ALL.

**Figure 1:**
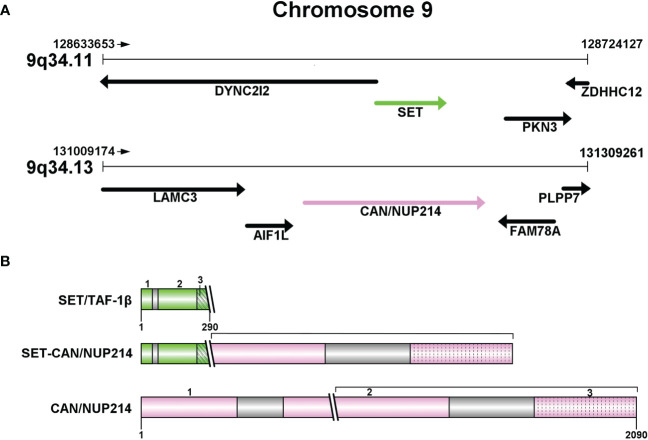
**(A)** Partial structure of chromosome 9 long arm (9q34): SET at 9q34.11 and CAN/NUP214 at 9q34.13. **(B)** Protein structures of SET/TAF-1β, CAN/NUP214 and SET-CAN/NUP214. SET/TAF-1β: 1-3: N-terminal dimerization domain; “Earmuff” domain; acidic and negatively charged C-terminal domain. CAN/NUP214: 1-3: β-propeller; coiled-coil region; FG repeats C-terminal region.

The NUP214 protein, also known as CAN, is a nucleoporin with FG repeats rich in phenylalanine-glycine. The *NUP214* gene is located on band 9q34.1 and it has a total of 36 exons numerically labeled from 1 to 36 (**Figure 1**). Chromosome abnormality involving *NUP214* occur repeatedly in leukemia, in addition to the *SET-CAN/NUP214* reviewed here, other chromosome abnormalities were found such as *DEK-NUP214*, *SQSTM1-NUP214* and *NUP214-ABL1*. *DEK-NUP214* [t(6;9)(p22;q34)] was associated with AML, *NUP214-ABL1* was identified in T-ALL patients, the rarest leukemia NUP214 fusion protein is SQSTM1-NUP214: to date, only two cases have been reported, one in ALL and the other in AML. The structure of the *SQSTM1-NUP214* fusion gene consists of five exons located at the N-terminus of the *SQSTM1* gene fused to a portion of the C-terminus of *NUP214*, including its last 14 FG repeats (7). In eukaryotic cells, nucleo-cytoplasmic transport plays an important role in maintaining the normal function and integrity of cells (8). Molecules with a molecular mass greater than 40kDa cannot move across the nuclear membrane by simple diffusion, but require to be facilitated by nuclear transporter receptors (NTRs) with the help of nuclear pore complexes (NPCs) embedded within the nuclear membrane (9–11). NUP214 interacts with NTRs via the FG repeat region in the cytoplasmic filaments of the nuclear pore complexes (NPCs) to control macromolecule trafficking (12). NUP214 has been shown to interact with exportin-1 (XPO1) and nuclear RNA export factor 1 (NXF1) of NTRs, which are highly mobile in cells (13) and play an important role in the response to NUP214 by nuclear export sequences (NES) protein; Furthermore, NUP214 fusion proteins such as SET-CAN/NUP214 and DEK-NUP214, reduce the mobility of XPO1 and lead to the accumulation of XPO1 cargo within the nucleus, impair nuclear output by sequestering XPO1 in the nucleus, interfere with nuclear-cytoplasmic transport of macromolecules, and potentially affect the transcriptional regulatory function of the NF-κB pathway (14), leading to various blood diseases (15). Moreover, genomic knockout of *NUP214* led to embryonic lethality in mice (1).

*SET*, also referred to as TATA box binding protein-associated factor 1 (*TAF1*). *SET* is a component of the histone acetyltransferase inhibitor (Inhat), which has been reported to be a putative oncogene involved in transcription by regulating chromatin organization (16). *SET* encodes a protein which can exert an inhibitory effect on apoptosis induced by cytotoxic T lymphocytes (4). In eukaryotic cells, the occurrence of selective splicing in the first two exons of the *TAF1* gene results in the formation of two forms of *SET* expression: the two heterodimeric forms, *TAF1-α* and *TAF1-β (* 1). Whereas in *SET-CAN/NUP214*, only the *TAF1-β* isoform is present (17). The structure of SET/TAF1-β consists of three parts: an N-terminal dimerization domain, a central “Earmuff” domain named for its headphone-like structure, and an acidic and negatively charged C-terminal domain (**Figure 1**). *SET/TAF-Iβ* has a variety of different activities, such as inhibiting phosphatase 2A activity, inducing cell transformation and differentiation, and transferring histones to naked DNA. The structural and negative regulatory functions may be related to glucocorticoid resistance (16, 18, 19).

*SET-CAN/NUP214* fusion gene encodes a protein containing an almost complete portion of SET fused to the carboxy-terminal two-thirds of CAN, which is a rare gene rearrangement occurs primarily in hematological malignancies (3). The appearance of the fusion gene may be the result of prior cancer therapy, but it may also occur *de novo*.

*SET-CAN/NUP214* positive patients often show resistance to chemotherapy including glucocorticoids, but the mechanism is not completely clear. The optional treatment has not been determined, previous studies have adopted different treatment options with varying prognoses for patients. Some previous studies have shown that *SET-CAN/NUP214* fusion gene positive patients have a worse prognosis (3, 7, 20), while clinical studies have shown that there is no significant difference in 3-year event-free survival (EFS) and overall survival (OS) between patients with *SET-CAN/NUP214* fusion gene positive and *SET-CAN/NUP214* negative patients (21, 22). Conventional techniques such as chromosomal karyotype analysis may have limitations in detecting patients with *SET-CAN/NUP214*. Due to the emergence of more advanced detection techniques such as fluorescence in situ hybridization (FISH), previously challenging fusion genes like *SET-CAN/NUP214* can now be detected with increasing frequency. This necessitates more precise disease classification and optimization of therapeutic regimens. Research shows that HSCT can improve the prognosis, the level of *SET-CAN/NUP214* after transplantation can predict recurrence to a certain extent (23), new methods such as CAR-T may be effective for patients and further research is needed (24).

In this review, we summarized the general features and clinical advances of *SET-CAN/NUP214* fusion gene in leukemia.

## 2 Materials and methods

### 2.1 Literature search

The cases and literature cited and included in this review were retrieved by Jingyu Song and his colleagues using PubMed, Web of Science, Google Scholar, and metstr databases or websites.

**Figure 2:**
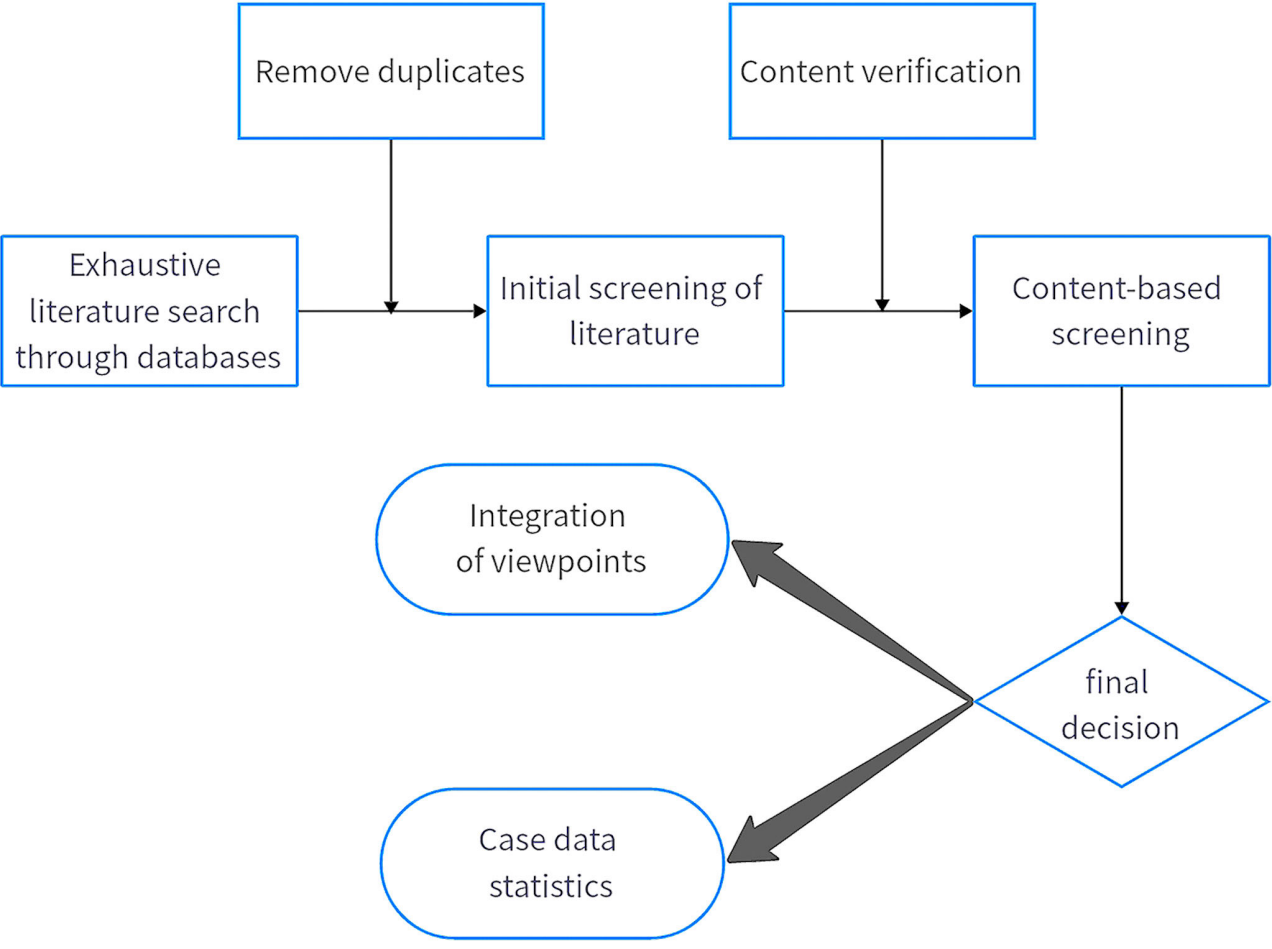
Flowchart for searching and selecting literature.

The whole screening process is shown in **Figure 2**. First we exhaustively searched the literature through the databases or websites, and in this step of the search we disregarded the country of publication and time constraints of the literature in order to obtain more comprehensive results. After the search was completed, we performed the exclusion of duplicates and initial screening. Next, by scanning the full-text content, we screened the literature based on its content and excluded incomplete and missing information, leaving behind content that (1) contained complete information and data (2) related to clinical cases, basic research, or reviews of *SET-CAN/NUP214*.

After completing the screening, we proceeded to the integration of viewpoints and statistics of cases.

### 2.2 Data analysis

We analyzed the statistical case data by SPSS software and performed survival analysis using Kaplan-Meier survival curves.

## 3 General features of the patient

In the 2022 international consensus classification of acute lymphoblastic leukemia/lymphoma, *SET-CAN/NUP214* fusion gene positive has been listed as a subtype of the *HOXA* gene family in the latest eight temporary entities (25). *SET-CAN/NUP214* fusion gene is rare in leukemia patients, and there is no prospective clinical study for such patients. Relevant articles focus on case reports and mechanism studies. This section provides an overview of the general features of patients.

According to a statistic in 2016, a total of 42 *SET-CAN/NUP214* positive patients were reported up to that year, including T-ALL(38/42,90.5%), AUL(2/42,4.8%), AML(1/42;2.4%) and B-ALL(1/42;2.4%) (4), another study involving 59 T-ALL patients showed that about 10.3% of T-ALL patients carried *SET-CAN/NUP214* fusion gene (20), Ben Abdelli et al. reported that the positive rate of *SET-CAN/NUP214* fusion gene in 196 patients with T-ALL was about 5.6% (21), in 2022, Yan C and others first reported two CML patients with positive *SET-CAN/NUP214* fusion gene (7). The data revealed that although *SET-CAN/NUP214* fusion gene occurs in various types of leukemia, it mainly occurs in T-ALL. This review compiled relevant literature containing more complete patient characteristics published since the emergence of the first *SET-CAN/NUP214* fusion gene positive case to date, some articles were not included due to lack of patient information, a total of 81 patients’ information was collected, the overall statistical characteristics of the patients are listed in **Table 1**, and detailed information on the individual characteristics of the patients are listed in **Table 2**. Among the 81 patients in **Table 2**, there are 57(57/81, 70.4%) patients with T-ALL, which is much higher than other types, consistent with the conclusion that the fusion gene is more likely to occur in T-ALL.

**Table 1:** Patient characteristic statistics.

Characteristics	Statistical overview
Age (year,range)	
average age	30.2 (8-58)
median age	29.0 (8-58)
Sex (n,%)	
male	59/81 (72.8%)
female	22/81 (27.2%)
Average WBC (×10^9^/L)	65.6
Subtype (n,%)	
T-ALL	57/81 (70.4%)
AML	12/81 (14.8%)
B-ALL	4/81 (4.9%)
MPAL	3/81 (3.7%)
AUL	2/81 (2.5%)
CML	2/81 (2.5%)
MS	1/81 (1.2%)
Treatment (n,%)	
Chemotherapy	20/69 (29.0%)
Transplant	49/69 (71.0%)
Clinical outcome (n,%)	
CR	52/69 (75.4%)
Relapse	28/69 (40.6%)
Death	30/69 (43.5%)

**Table 2:** Characteristics of SET-CAN/NUP214 positive patients reported in the literature.

Case no.	Diagnosis	Year	Ref.	Sex	Age (y)	WBC(×10^9^/L)	Immunophenotype/ Flow cytometry					
							CD7	CD33	CD34	CD13	cCD3	
1	AUL	1992	Von Lindern (17)	Male	19.0	/	+	+	–	–	–	
2	AUL	2010	Kim.J (26)	Male	40.0	53	+	+	–	–	+	
3	MS	2020	Zhang.H (6)	Female	32.0	4.15	+	+	–	–	–	
4	MPAL	2020	Li MY (27)	Male	29.0	0.56	+	+	+	–	+	
5	MPAL	2021	Chen SM (28)	Female	22.0	/	/	/	/	/	/	
6	MPAL	2021	Chen SM (28)	Male	34.0	/	/	/	/	/	/	
7	AML	2007	Rosati R (29)	Male	35.0	40	–	+	+	+	–	
8	AML	2019	Jeong IH (30)	Male	46.0	17.1	+	+	+	–	–	
9	AML	2020	Zhang.H (6)	Male	24.0	11.41	+	+	+	+	–	
10	AML	2021	Zheng YZ (31)	Male	12.0	231.8	+	+	+	+	–	
11	AML	2021	Zheng YZ (31)	Male	10.0	38.75	+	+	+	+	–	
12	AML	2021	Chen SM (28)	Male	20.0	/	/	/	/	/	/	
13	AML	2021	Chen SM (28)	Male	32.0	/	/	/	/	/	/	
14	AML	2021	Chen SM (28)	Male	26.0	/	/	/	/	/	/	
15	AML	2021	Chen SM (28)	Male	12.0	/	/	/	/	/	/	
16	AML	2021	Chen SM (28)	Female	46.0	/	/	/	/	/	/	
17	AML	2021	Chen SM (28)	Male	38.0	/	/	/	/	/	/	
18	AML	2021	Chen SM (28)	Male	50.0	/	/	/	/	/	/	
19	CML	2022	Chen Y (5)	Male	42.0	/	–	+	+	+	–	
20	CML	2022	Chen Y (5)	Female	37.0	283.5	–	+	+	+	–	
21	B-ALL	2010	Nowak NJ (32)	Female	42.0	/	/	/	/	/	/	
22	B-ALL	2014	Hong HZ (4)	Male	19.0	217.0	+	+	+	+	–	
23	B-ALL	2021	Chen SM (28)	Male	18.0	/	/	/	/	/	/	
24	B-ALL	2021	Chen SM (28)	Male	22.0	/	/	/	/	/	/	
25	T-ALL	2008	Van Vlierberghe P (33)	Female	15.3	213.0	/	/	/	/	/	
26	T-ALL	2008	Van Vlierberghe P (33)	Female	10.6	142.0	/	/	/	/	/	
27	T-ALL	2008	Van Vlierberghe P (33)	Female	17.1	15	/	/	/	/	/	
28	T-ALL	2010	Gorello P (7)	Male	38.0	24	/	/	/	/	/	
29	T-ALL	2010	Gorello P (7)	Male	19.0	3.28	/	/	/	/	/	
30	T-ALL	2010	Gorello P (7)	Male	47.0	/	/	/	/	/	/	
31	T-ALL	2010	Gorello P (7)	Female	27.0	/	/	/	/	/	/	
32	T-ALL	2010	Gorello P (7)	Male	19.0	/	/	/	/	/	/	
33	T-ALL	2010	Gorello P (7)	Male	18.0	/	/	/	/	/	/	
34	T-ALL	2010	Gorello P (7)	Male	23.0	/	/	/	/	/	/	
35	T-ALL	2011	Lee SG (34)	Male	28.0	37.3	+	+	+	–	–	
36	T-ALL	2011	Chae H (35)	Female	55.0	24.43	+	+	+	+	+	
37	T-ALL	2011	Chae H (35)	Female	32.0	18.04	+	+	+	+	+	
38	T-ALL	2011	Chae H (35)	Male	32.0	39.06	+	+	+	–	+	
39	T-ALL	2011	Chae H (35)	Male	20.0	5.07	+	+	+	–	+	
40	T-ALL	2011	Li WJ (36)	Female	12.0	1.5	+	+	+	+	+	
41	T-ALL	2011	Li WJ (36)	Male	11.0	6.4	+	+	–	–	+	
42	T-ALL	2011	Li WJ (36)	Male	8.0	99.6	+	–	+	–	+	
43	T-ALL	2012	Dai HP (20)	Male	20.0	34.1	+	+	+	+	+	
44	T-ALL	2012	Dai HP (20)	Female	56.0	6.8	+	+	+	–	+	
45	T-ALL	2012	Dai HP (20)	Female	23.0	2.6	+	+	+	–	+	
46	T-ALL	2012	Dai HP (20)	Male	27.0	/	+	+	+	+	+	
47	T-ALL	2012	Dai HP (20)	Male	45.0	33.3	+	+	+	–	+	
48	T-ALL	2012	Dai HP (20)	Male	23.0	15.1	+	+	+	–	+	
49	T-ALL	2012	Lee EY (37)	Female	43.0	60.6	+	+	+	+	+	
50	T-ALL	2014	Ben (21)	Male	34.0	30.4	+	+	+	–	+	
51	T-ALL	2014	Ben (21)	Female	37.0	8.6	+	–	+	–	+	
52	T-ALL	2014	Ben (21)	Male	29.0	10.1	+	+	+	+	+	
53	T-ALL	2014	Ben (21)	Male	41.0	18.4	+	+	+	–	+	
54	T-ALL	2014	Ben (21)	Male	23.0	604.4	+	–	–	–	+	
55	T-ALL	2014	Ben (21)	Male	30.0	24.9	+	–	–	–	+	
56	T-ALL	2014	Ben (21)	Male	36.0	181.8	+	+	+	–	+	
57	T-ALL	2014	Ben (21)	Male	45.0	50.8	+	–	–	–	+	
58	T-ALL	2014	Ben (21)	Male	38.0	2.8	+	+	+	–	+	
59	T-ALL	2014	Ben (21)	Male	28.0	41.8	+	+	+	–	+	
60	T-ALL	2014	Ben (21)	Male	20.0	30.9	+	–	–	–	+	
61	T-ALL	2015	Prokopiou C (38)	Female	48.0	/	+	–	+	–	+	
62	T-ALL	2015	Prokopiou C (38)	Male	45.0	/	+	+	+	–	–	
63	T-ALL	2019	Yang Q (3)	Male	26.0	12.3	+	–	–	–	–	
64	T-ALL	2019	Yang Q (3)	Male	51.0	109.1	+	+	–	–	–	
65	T-ALL	2019	Yang Q (3)	Male	37.0	131.5	+	+	+	–	–	
66	T-ALL	2020	Zhang.H (6)	Male	21.0	37.16	–	–	–	–	–	
67	T-ALL	2021	Xianying Xu (39)	Female	44.0	21.1	+	–	+	–	+	
68	T-ALL	2021	Na Lin (40)	Female	15.0	23.5	+	–	+	–	+	
69	T-ALL	2021	Chen SM (28)	Male	58.0	/	/	/	/	/	/	
70	T-ALL	2021	Chen SM (28)	Female	27.0	/	/	/	/	/	/	
71	T-ALL	2021	Chen SM (28)	Male	37.0	/	/	/	/	/	/	
72	T-ALL	2021	Chen SM (28)	Male	27.0	/	/	/	/	/	/	
73	T-ALL	2021	Chen SM (28)	Female	16.0	/	/	/	/	/	/	
74	T-ALL	2021	Chen SM (28)	Male	36.0	/	/	/	/	/	/	
75	T-ALL	2021	Chen SM (28)	Male	40.0	/	/	/	/	/	/	
76	T-ALL	2021	Chen SM (28)	Male	41.0	/	/	/	/	/	/	
77	T-ALL	2021	Chen SM (28)	Female	34.0	/	/	/	/	/	/	
78	T-ALL	2021	Chen SM (28)	Male	15.0	/	/	/	/	/	/	
79	T-ALL	2021	Chen SM (28)	Male	12.0	/	/	/	/	/	/	
80	T-ALL	2021	Chen SM (28)	Male	42.0	/	/	/	/	/	/	
81	T-ALL	2021	Chen SM (28)	Male	36.0	/	/	/	/	/	/	

AUL, Acute undifferentiated leukemia; ALL, Acute lymphoblastic leukemia; T-ALL, T-cell ALL; B-ALL, B-cell ALL; AML, Acute myeloid leukemia; MS, Myeloid sarcoma; MPAL, Mixed phenotype acute leukemia; CML, Chronic myeloid leukemia; Immunophenotype positive: +; Immunophenotype negative: -; unknown:/

Among the fusion gene positive patients counted in this review, there are 59 male and 22 female patients, respectively, with the proportion of male patients reaching more than 70%, suggesting that the *SET-CAN/NUP214* fusion gene is more likely to occur in male patients. The number of fusion gene positive T-ALL patients included 40 males and 17 females, with the proportion of males reaching 70.2%. Although there were fewer cases of other types of leukemia, there were still significantly more males than females, which suggests that the type of leukemia in fusion gene positive patients may not be an influencing factor in the proportion of males and females in the disease (4, 7, 20, 21).

There is a large difference in the age of patients at initial diagnosis, the youngest patient is only 8 years old (T-ALL), the oldest patient is 58 years old (T-ALL), the average age is 30.2 years old and the patients are distributed in all age groups (6, 17, 24, 26, 35, 41). Relatively speaking, the probability of fusion gene positive in adult leukemia patients is higher (40). Two CML patients with *SET-CAN/NUP214* fusion gene positive were 37 and 42 years old, far from the average age of fusion gene positive patients. However, due to the small number of cases and the older age of CML patients, the relationship between age and fusion gene could not be established.

In previous cases, the patients with fusion gene positive leukemia did not show symptoms different from those with fusion gene negative leukemia, and most remained symptomatic with classic anemia, fever, and lower sternal segment tenderness. However, liver and spleen enlargement, lymph node enlargement, mediastinal involvement, as well as tumor bulk and rapid growth were more common than in fusion gene negative patients (5, 29, 34, 42). Some patients came to see doctors because of liver and spleen enlargement and related symptoms caused by mediastinal mass. Sang-Guk Lee et al. described a 28-year-old patient who complained of dyspnea and chest pain. Physical examination found that multiple lymph nodes in the neck were swollen. Chest CT showed that mediastinal mass compressed the main pulmonary artery with pleural effusion and splenomegaly. Finally, the patient was diagnosed as *SET-CAN/NUP214* positive T-ALL (34). Song Y et al. (43) also confirmed that patients often have extramedullary infiltration at the onset of the disease, including areas such as the skin, liver and breast. According to the statistics, the median WBC count of the patients was 18.0×10^9^/L. Based on the collected patient information, the highest WBC count was 604.4 × 10^9^/L (T-ALL) and this patient died 5 months after diagnosis. The median percentage of leukemic blasts in the bone marrow was high (82.0-97.0%), probably reflecting the high proliferation status of fusion gene positive patients (4, 24).

Patients with fusion gene positive may have normal chromosome karyotype or complex karyotype, the existence of a complex karyotype may mask the presence of the fusion gene (34, 44). As a molecular abnormality with low frequency, this is also the reason why *SET-CAN/NUP214* patients were not widely concerned at first.

In terms of immunophenotype, the fusion gene positive leukemia cells showed characteristics of extreme immaturity. Flow cytometry showed that their most frequent immunophenotype was CD7, except for the two CML cases mentioned previously (7), only one T-ALL patient and one AML patient reported by Zhang H (6)and Rosati R (29) did not detect CD7+. CD7 was highly frequent in *SET-CAN/NUP214* fusion gene positive leukemia, and the other immunophenotypes with higher frequency were cCD3, CD34, CD33 and CD13. The immunophenotypic results suggest that the transformation of fusion gene positive leukemia may occur in the early stage of myeloid or T-lymphocyte differentiation, and it may be related to the inhibition of differentiation of primitive progenitor cells by the fusion gene (6, 7, 35, 45, 46).

Generally, myeloid markers such as CD13 and CD33 are only expressed in about 19% of T-ALL cases. The reason why the fusion gene induces myeloid marker expression remains to be further investigated.

## 4 Molecular anomaly in SET-CAN/NUP214

*SET-CAN/NUP214* fusion gene impairs the process of hematopoietic differentiation, but it alone is not sufficient to induce leukemia. Additional chromosomal aberrations and molecular events are required to mediate the development of leukemia. Understanding the process is greatly helpful for understanding the disease.

*SET-CAN/NUP214* fusion gene may contribute to leukemia through direct and indirect effects. Saito S et al. (47) developed transgenic mice expressing *SET-CAN/NUP214*, which is active in different groups of hematopoietic cell groups, and the transgenic mice carrying *SET-CAN/NUP214* gradually developed symptoms such as anemia, thrombocytopenia and splenomegaly, so that within 6 months, a considerable number of transgenic mice died successively, the course and characteristics of the lesions are more similar to those of leukemias, and the characterization of bone marrow cells in mice during the course of the disease showed that the *SET-CAN/NUP214* fusion gene increased the number of immature cells and impaired the hematopoietic differentiation of erythroid, granulocytic, and megakaryocytic lineages (47).

Previous studies have shown that the fusion gene impairs the process of hematopoietic differentiation, but cannot induce the occurrence of leukemia alone. *HOXA* upregulation may be the key mechanism and play an intermediary role. *HOX* genes is a kind of gene that specially regulates biological form in organisms. The expression of *HOX* gene in various organisms is similar, and its sequence is related to its action sequence and action position. Human *HOX* gene can be divided into four gene clusters: *HOXA*, *HOXB*, *HOXC*, and *HOXD*, which are located on different chromosomes respectively. The DNA sequence of these gene family members is similar to the protein sequence transcribed. Quantitative and comparative analysis of bone marrow samples from *SET-CAN/NUP214* positive patients during initial diagnosis and morphological remission using RT-PCR and other detection methods showed that the expression level of *HOXA9* and *HOXA10* at initial diagnosis was 3.53 and 4.15 times higher than that during morphological remission, while the expression level of *HOXA5* was similar (34). Sang-Guk Lee and others found that the up-regulation of *HOXA* gene was caused by the interaction of *SET-CAN/NUP214* fusion gene with *XPO1*, *hDOT1L* and *HOXA* promoters. The fusion genes with similar mechanism include *CALM-AF10* and *MLL-AF10 (* 33), which can lead to the H3K79 hypermethylation of *HOXA* genes and mediate the occurrence of leukemia. Hypermethylation and subsequent upregulation of *HOXA* genes play an important role in the pathogenesis of leukemia with positive fusion gene (48). Gorello P et al. detected the overexpression of *HOXA7*, *HOXA9* and *HOXA10 in the* fusion gene positive patients selected from 256 ALL patients (7, 42). 17 fusion gene positive patients were detected by FISH analysis, and all patients(17/17, 100%) had overexpression of *HOXA* gene. There were also studies that summarized the up-regulation of *HOXA* gene with the positive expression of several *NUP214* fusion gene subtypes. *SET-NUP214*, *DEK-NUP214* and *SQSTM1-NUP214* have the same characteristics, which can lead to the up-regulation of *HOXA3*, *HOXA5*, *HOXA7*, *HOXA9*, *HOXA10* and *HOXB* in the *HOX* family (2, 33, 49, 50). These studies also confirmed the relationship between *HOXA* and *SET-CAN/NUP214* fusion gene.

Na Lin et al. (40) evaluated common recurrent mutations in *SET-CAN/NUP214* positive T-ALL patients through next-generation sequencing. The results showed that mutations were more common in *NOTCH1*(23/31,74.2%), *PHF6*(11/21,52.38%), *KRAS*(6/14,42.86%), *JAK3*(4/12,33.33%), *CCND3*(3/12,25%), *JAK1*(3/15,20%), *STAT5B*(2/10,20%), *DNM2*(2/10,20%) and *EED*(2/10,20%), these are common recurrent mutations in *SET-CAN/NUP214* positive patients in T-ALL and ETP-ALL. The patients with fusion gene positive are accompanied by more molecular events than those with fusion gene negative. These complex molecular events may promote adverse reactions to induction therapy, and may also be one of the factors of poor prognosis (51). As the total number of cases remains low, these issues remain to be explored.

The protein encoded by *NOTCH* gene is a highly conserved cell surface receptor, which can regulate the development of a variety of biological cells. *NOTCH* signaling can affect a series of normal life processes of cells, including the differentiation of pluripotent progenitor cells, cell apoptosis, proliferation and cell boundary formation. The abnormality of *NOTCH* signaling is related to esophageal cancer, gastric cancer, leukemia and other diseases, Among them, abnormal *NOTCH1* is most often detected in tumor diseases.

The activation mutation of *NOTCH1* or the inactivation mutation of *NOTCH1* negative regulatory factor(*FBXW7*) can be found in about 60% of T-ALL cases. However, the proportion of *NOTCH1* mutation seems to be higher in *SET-CAN/NUP214* positive leukemia patients. A gene sequencing of 6 *SET-CAN/NUP214* positive T-ALL patients by Dai HP et al.(Jiangsu Institute of Hematology, China) showed most T-ALL patients with positive fusion gene have *NOTCH1* mutations(5/6,83.3%) and *PHF6* mutations(4/6,66.7%) (20). The next-generation sequencing of patients by Na Lin et al. (40) showed that the proportion of *NOTCH1* mutations in 31 patients reached 74.2%, similarly, the results of the test performed by Wang Q et al. (52) on the association between 96 fusion gene positive patients and mutations such as *NOTCH*, *JAK1* and others demonstrated a possible positive correlation between *NOTCH1* mutations and fusion gene positivity.

The mutations of *NOTCH1*, *PHF6* and *JAK1* are closely linked in the process of leukemia, which may be the secondary genetic alterations of *SET-CAN/NUP214* fusion gene. *PHF6* is a tumor suppressor gene with transcriptional regulation linked to the X sex chromosome in the nucleus. Tumorigenic mutations have a higher incidence rate in T-ALL and can also be seen in AML, most of them occur in male patients. *JAK1* plays a key role in initiating reactions related to a variety of major cytokine receptor families. It appears in about 20% of adult T-ALL patients, generally indicating poor prognosis. If the patients with positive fusion gene have co-mutation of *NOTCH1* and *PHF6*, they are more likely to have symptoms such as splenomegaly and lymph node enlargement (2, 22, 52–54). In addition, the existence of *SET-CAN/NUP214* fusion gene is related to the up-regulation of the expression level of lymphoblastic leukemia-associated hematopoietic regulator 1(*LYL1*) and myocyte enhancer 2C(*MEF2C*) genes (22). Contrary to the common mutations such as *NOTCH1*, *PHF6* and *JAK1*, the overexpression of *CALM-AF10*, *SIL-TAL*, *TLX1* or *TLX3* is mutually exclusive with the existence of *SET-CAN/NUP214* fusion gene. A gene test of 11 fusion gene positive T-ALL patients by Ben et al (21). showed that none of the 11 patients expressed *CALM-AF10*, *SIL-TAL*, *TLX1* or *TLX3*(0/11,0%).

In the process of leukemogenesis mediated by *SET-CAN/NUP214* fusion gene, it is generally accepted that additional chromosomal aberrations also play a role. Chae H et al. (35) reported del (12)(p13)/ETV6 in 3 of 4 patients, while Ben et al. (21) found this aberration numerous times in their cases. Similarly, the patients in the reports also presented del (6) (q21q23) and del (11) (q22q23) chromosomal aberrations (51, 55). The recurrent chromosomal aberrations in the rare fusion gene positive patients are intriguing and worth pondering.

## 5 Treatment and prognosis of patients

### 5.1 Prognosis of leukemia patients with SET-CAN/NUP214 fusion gene

The prognosis of patients with positive *SET-CAN/NUP214* fusion gene is different. Most studies consider that the prognosis is poor. The prognosis of patients may vary due to leukemia classification, concomitant molecular events, treatment plan and the age stage. Patients generally showed delayed response and drug resistance to chemotherapy including glucocorticoids, but studies showed that this drug resistance might not have a negative impact on clinical outcomes (21). Yang Q et al. demonstrated that the prognosis of T-ALL patients with *SET-CAN/NUP214* was quite poor, their treatment of three patients with fusion gene positive showed that none of the three patients achieved complete remission(CR) during chemotherapy, and all of them were infected by drug-resistant bacteria such as Candida tropicalis and Pseudomonas aeruginosa. Because of the disease progress and the inability to control the concurrent infection, two patients died during chemotherapy (3). Gorello P et al. also found that the prognosis of fusion gene positive patients was poor. In this study, 6 of the 7 patients received treatment, of which 4 patients died 12 to 24 months after treatment. The main causes of death were refractory disease and leukemia recurrence (7). The treatment results of 6 patients by Dai HP et al. showed that 4 of the 6 patients had recurrence (the median recurrence time was only 7.8 months), and 3 of them died (20). There are also studies show that the positive fusion gene has no effect on the clinical outcome of patients. In the study of Ben et al., the difference between the 3-year total survival rate(3y OS) and event-free survival rate(3y EFS) of fusion gene positive patients and fusion gene negative patients is not statistically significant(3y OS:73% vs 68%; 3y EFS:45% vs 59%) (21, 24), while in the study of Chen B et al, the 3-year overall survival rate(3y OS) and event-free survival rate(3y EFS) of 8 fusion gene positive patients were 87.5% and 70% respectively (22). It can be seen that the outcomes of patients in different clinical trials vary greatly, and finding more effective treatment methods may be beneficial to patients.

### 5.2 Studies on the causes of corticosteroid resistance

Patients with positive *SET-CAN/NUP214* fusion gene usually exhibit general resistance to chemotherapy regimens including glucocorticoids in the early stages of induction therapy. Although patients have a delayed response to chemotherapy, the overall CR rate is not affected (40).

The relevant research evaluated patients based on *in vitro* drug sensitivity screening, monitoring of blasts during induction and MRD results after induction. Compared with the patients with negative fusion gene, the rate of corticosteroid resistance in patients with positive fusion gene(91% of patients had corticosteroid resistance, while the data of patients with negative fusion gene was only 44%) and the rate of early chemotherapy resistance (nearly 100% of patients had early chemotherapy resistance, and only 44% of patients with negative fusion gene) were significantly higher (3, 34, 39, 40).

The anti-inflammatory, immunosuppressive and proapoptotic effects of glucocorticoids play an important role in the treatment of various inflammatory, autoimmune and tumor diseases. In the treatment of leukemia, glucocorticoids are involved in various chemotherapy regimens, especially for ALL. Corticosteroid therapy induced GR target gene transcription is also one of the reference treatment options for ALL (56, 57). The powerful role of glucocorticoids is based on the ubiquitous glucocorticoid receptors(GR) in human cells (58), ligands activate GR and bind with glucocorticoid response elements(GREs) in the nucleus. The transcription process starts under the mediation of “coactivators” such as steroid receptor coactivator 1 (*SRC1*) and glucocorticoid receptor interaction protein 1 (*GRIP1*). Under pathological conditions, *SET* is fused with *CAN/NUP214*, and the *SET* subtype mainly exists in *SET-CAN/NUP214* is *TAF1-β*. *TAF1-β* serves as a component in the INHAT complex, which interacts with a variety of trans-acting factors through *TAF1-β* to inhibit the transcriptional activity of multiple transcription factors and nuclear receptors. Due to this mechanism, Takamasa Ichijo et al. reported that the potential cause of glucocorticoid resistance in patients with positive *SET-CAN/NUP214* fusion gene is the co-precipitation of SET-CAN/NUP214 fusion protein and glucocorticoid response element, which inhibits the transcription activity of glucocorticoid receptor and histone acetylation (56, 59). The *in vitro* experimental data reported by Yang Q and others also believe that the lack of histone acetylation regulation mediated by *SET-CAN/NUP214* may be the cause of glucocorticoid resistance in many patients (3).

Even though nearly 100% of *SET-CAN/NUP214* fusion gene positive patients exhibit resistance during the early stages of chemotherapy, studies have shown a high complete response rate(26 of 36 patients,72.22%) (40, 60). The CR rate of the 69 patients counted in **Table 1** is also relatively high, reaching 75.4% (52/69). The drug resistance situation and mechanism of the patients still need further research, which may be helpful for the selection of chemotherapy regimen.

### 5.3 Chemotherapy and transplantation

The optional treatment method of *SET-CAN/NUP214* fusion gene positive leukemia has not been determined. We present patients with clear treatment methods and outcome information reported so far in **Table 3** for reference. Analyzing the treatment methods and prognosis of previous cases may provide guidance for the establishment of treatment strategies for such patients.

**Table 3:** Treatment and outcome of patients.

Case no.	Diagnosis	Year	Ref./Year	Treatment	Outcome
1	AUL	40	Kim.J (26) 2010	cytosine arabinoside, idarubicin	CR, alive 7 months and lost to follow-up
2	MS	32	Zhang.H (6) 2020	idarubicin, cytarabine homoharringtonine	Myelosoppression with a rapidly increased pericardial effusion
3	MPAL	29	Li MY (27) 2020	idarubicin, vincristine, dexamethasone, hyper-CVAD-A regimen, hyper-CVAD-B regimen, HSCT,CAR-T	Chemotherapy achieved CR, HSCT,CAR-T,relapse alive>42 months
4	MPAL	22	Chen SM (28) 2021	CODLP or VPIA(vincristine + prednisone + daunorubicin + cytarabine) HSCT	CR,HSCT alive>42 months
5	MPAL	34	Chen SM (28) 2021	CODLP or VPIA(vincristine + prednisone + daunorubicin + cytarabine) HSCT	CR,HSCT alive>24 months
6	AML	35	Rosati R (29) 2007	daunorubicin, cytosine arabinoside HSCT	CR,HSCT still alive
7	AML	46	Jeong IH (30) 2019	idarubicin and cytosine arabinoside,HSCT	CR,HSCT still alive
8	AML	24	Zhang.H (6) 2020	daunorubicin,cytarabine HSCT	CR,HSCT alive>8 months
9	AML	12	Zheng YZ (31) 2021	Cytarabine,FLAG-IDA allo-HSCT	CR,HSCT relapse,died +16.5months
10	AML	10	Zheng YZ (31) 2021	FLAG-IDA allo-HSCT	CR,HSCT alive>27 months
11	AML	20	Chen SM (28) 2021	combination chemotherapy HSCT	CR,HSCT alive>34 months
12	AML	32	Chen SM (28) 2021	combination chemotherapy HSCT	CR,HSCT alive>40 months
13	AML	26	Chen SM (28) 2021	combination chemotherapy HSCT	CR,HSCT relapse alive>90 months
14	AML	12	Chen SM (28) 2021	combination chemotherapy HSCT	CR,HSCT alive>32 months
15	AML	46	Chen SM (28) 2021	combination chemotherapy HSCT	CR,HSCT alive>41 months
16	AML	38	Chen SM (28) 2021	combination chemotherapy HSCT	CR,HSCT relapse, died +45months
17	AML	50	Chen SM (28) 2021	combination chemotherapy HSCT	CR,HSCT relapse, died +25months
18	CML	42	Chen Y (5) 2022	Imatinib, dasatinib, decitabine, venetoclax, ponatinib HSCT	partial response, HSCT alive>95.7 month
19	CML	37	Chen Y (5) 2022	Imatinib, dasatinib Idarubicin, cytarabine	Increased after two years of treatment with Imatinib, change to dasatinib, idarubicin and cytarabine relapse,died+36 months
20	B-ALL	18	Chen SM (28) 2023	combination chemotherapy HSCT	HSCT died +9months
21	B-ALL	22	Chen SM (28) 2023	combination chemotherapy HSCT	CR,HSCT relapse, died +15months
22	T-ALL	38	Gorello P (7) 2010	combination chemotherapy ASCT	CR, ASCT alive>29 months
23	T-ALL	19	Gorello P (7) 2010	/	CR, SCT relapse, died +23months
24	T-ALL	27	Gorello P (7) 2010	/	drug resistance died +12months
25	T-ALL	19	Gorello P (7) 2010	/	CR alive>3 months
26	T-ALL	18	Gorello P (7) 2010	/	CR relapse, died +24months
27	T-ALL	23	Gorello P (7) 2010	combination chemotherapy ASCT	CR, ASCT relapse, died +17 months
28	T-ALL	55	Chae H (35) 2011	/	relapse alive>31 months
29	T-ALL	32	Chae H (35) 2011	/	relapse died +42 months
30	T-ALL	32	Chae H (35) 2011	/	relapse died +21 months
31	T-ALL	20	Chae H (35) 2011	HSCT	HSCT alive>41 months
32	T-ALL	12	Li WJ (36) 2011	allo-HSCT	Allo-HSCT relapse, alive
33	T-ALL	11	Li WJ (36) 2011	combination chemotherapy	died +10 months
34	T-ALL	8	Li WJ (36) 2011	combination chemotherapy	CR, alive
35	T-ALL	20	Dai HP (20) 2012	combination chemotherapy	CR, relapse died +9months
36	T-ALL	23	Dai HP (20) 2012	combination chemotherapy	CR relapse, alive>18months
37	T-ALL	27	Dai HP (20) 2012	combination chemotherapy	CR relapse, died +15months
38	T-ALL	45	Dai HP (20) 2012	combination chemotherapy	CR relapse, died +30months
39	T-ALL	34	Ben (21) 2014	GRAALL trail	CR, SCT relapse, died +49months
40	T-ALL	37	Ben (21) 2014	GRAALL trail	CR, SCT alive>64months
41	T-ALL	29	Ben (21) 2014	GRAALL trail	CR, SCT relapse, alive>44months
42	T-ALL	41	Ben (21) 2014	GRAALL trail	CR, SCT alive>46months
43	T-ALL	23	Ben (21) 2014	GRAALL trail	died +5months
44	T-ALL	30	Ben (21) 2014	GRAALL trail	CR, SCT relapse, alive>66months
45	T-ALL	36	Ben (21) 2014	GRAALL trail	CR, SCT alive>24months
46	T-ALL	45	Ben (21) 2014	GRAALL trail	CR alive>33months
47	T-ALL	38	Ben (21) 2014	GRAALL trail	SCT died +9months
48	T-ALL	28	Ben (21) 2014	GRAALL trail	CR, SCT alive>30months
49	T-ALL	20	Ben (21) 2014	GRAALL trail	CR, SCT alive>28months
50	T-ALL	48	Prokopiou C (38) 2015	combination chemotherapy	ASCT died +12months
51	T-ALL	45	Prokopiou C (38) 2015	combination chemotherapy	ASCT died +6months
52	T-ALL	26	Yang Q (3) 2019	VICP	died of infection +15days
53	T-ALL	51	Yang Q (3) 2019	VICP, mitoxantroned, etoposide, cytarabine	died of infection +37days
54	T-ALL	37	Yang Q (3) 2019	CALGB9111, CLAG, asparaginase	alive>10months
55	T-ALL	21	Zhang.H (6) 2020	VICP, hyper-CVAD-B, MTX, cladribine,decitabine, HSCT	CR, HSCT alive>14months
56	T-ALL	44	Xianying Xu (39) 2021	VDCLP, CAM(cyclophosphamide, cytosine arabinoside, 6-mercaptopurine), chidamide	CR, but the disease progressed again within a month
57	T-ALL	15	Na Lin (40) 2021	VICLP, methotrexate, pegaspargase, DAE, EAD,Hypr-CVAD-A/B, HSCT	CR, HSCT alive>16months
58	T-ALL	58	Chen SM (28) 2021	combination chemotherapy HSCT	CR,HSCT alive>35 months
59	T-ALL	27	Chen SM (28) 2021	combination chemotherapy HSCT	CR,HSCT relapse, died +24months
60	T-ALL	37	Chen SM (28) 2021	combination chemotherapy HSCT	CR,HSCT alive>59 months
61	T-ALL	27	Chen SM (28) 2021	combination chemotherapy HSCT	CR,HSCT relapse, died +26months
62	T-ALL	16	Chen SM (28) 2021	combination chemotherapy HSCT	CR,HSCT alive>41 months
63	T-ALL	36	Chen SM (28) 2021	combination chemotherapy HSCT	CR,HSCT relapse, died +15months
64	T-ALL	40	Chen SM (28) 2021	combination chemotherapy HSCT	CR,HSCT relapse, died +18months
65	T-ALL	41	Chen SM (28) 2021	combination chemotherapy HSCT	CR,HSCT died +22months
66	T-ALL	34	Chen SM (28) 2021	combination chemotherapy HSCT	CR,HSCT alive>51 months
67	T-ALL	15	Chen SM (28) 2021	combination chemotherapy HSCT	CR,HSCT relapse, died +12months
68	T-ALL	12	Chen SM (28) 2021	combination chemotherapy HSCT	CR,HSCT relapse, died +12months
69	T-ALL	42	Chen SM (28) 2021	combination chemotherapy HSCT	CR,HSCT alive>29 months
70	T-ALL	36	Chen SM (28) 2021	combination chemotherapy HSCT	CR,HSCT relapse, alive>14 months

The *SET-CAN/NUP214* fusion gene is mainly found in T-ALL patients. **Table 3** contains 49 T-ALL patients, of which 18 patients received chemotherapy and 31 patients received transplantation. Among the patients receiving chemotherapy, 7 patients survived, 11 patients died, and 9 patients relapsed; Among the patients receiving transplantation, 18 patients survived, 13 patients died and 13 patients relapsed.

Most patients developed drug resistance at the initial stage of chemotherapy, but 35 T-ALL patients finally achieved complete remission (CR, 35/49, 71.4%), which was similar to the complete remission rate suggested in previous studies (72.22%) (40, 60). Yang Q et al. (3) reported that CLAG chemotherapy combined with asparaginase might be a potential treatment option for adult *SET-CAN/NUP214* fusion gene positive T-ALL patients. They implemented VICP chemotherapy for the first two patients (No.52-53) in the case, but the effect was not obvious. The patients eventually died because of the disease progress and uncontrollable infection of drug-resistant bacteria, for the third patient(No.54), the CLAG chemotherapy regimen combined with asparaginase was used. Surprisingly, the patient’s condition was quickly controlled. Na Lin et al. (40) conducted a drug sensitivity screening tests on the leukemic cells of a refractory fusion gene positive T-ALL patient (No.57) with up to 165 drugs, suggesting that the DAE protocol of “AML like treatment” (daunorubicin+cytarabine+etoposide) showed the highest inhibition rate *in vitro*. At the same time, they suggested that the induction treatment could adopt a 28-day course of chemotherapy such as used in GRAALL 2003 or 2005. The reason why such “AML like treatment” is effective for patients with fusion gene positive may be related to the frequent occurrence of markers such as CD33 and CD34. Carfilzomib may have a strong inhibitory effect on leukemic cells with positive fusion gene. It can mediate the production of reactive oxygen species as an inducer and synergistically enhance the cytotoxicity of dexamethasone. It is worth noting that in the drug sensitivity screening test, the inhibition rate of single drug treatment of carfilzomib is 37.57%, which shows that carfilzomib may also have potential benefits for patients with refractory *SET-CAN/NUP214* fusion gene positive T-ALL (40, 61, 62). Unfortunately, carfilzomib is not currently available in China.

In the treatment of fusion gene positive patients, transplantation may benefit more. A literature based comparison of the treatment methods of patients shows that the average survival time of the chemotherapy group was 22.5 months, the average survival time of the transplantation group was 50.1 months, the average survival time of the chemotherapy group was less than half of that in the transplantation group (24). The statistical analysis shows that hematopoietic stem cell transplantation (HSCT) can significantly improve the survival rate of patients, we can consider that only chemotherapy for patients with fusion gene positive is not enough. The total 3-year overall survival rate (3y OS) of the 9 patients with fusion gene positive T-ALL who received allogeneic hematopoietic stem cell transplantation was 73% (21), which is similar to the outcome of the patients with fusion gene negative after allogeneic hematopoietic stem cell transplantation. This suggests that transplantation can significantly improve the prognosis of patients. It may be a good choice to complete the transplantation at the right time in the first CR.

**Figure 3:**
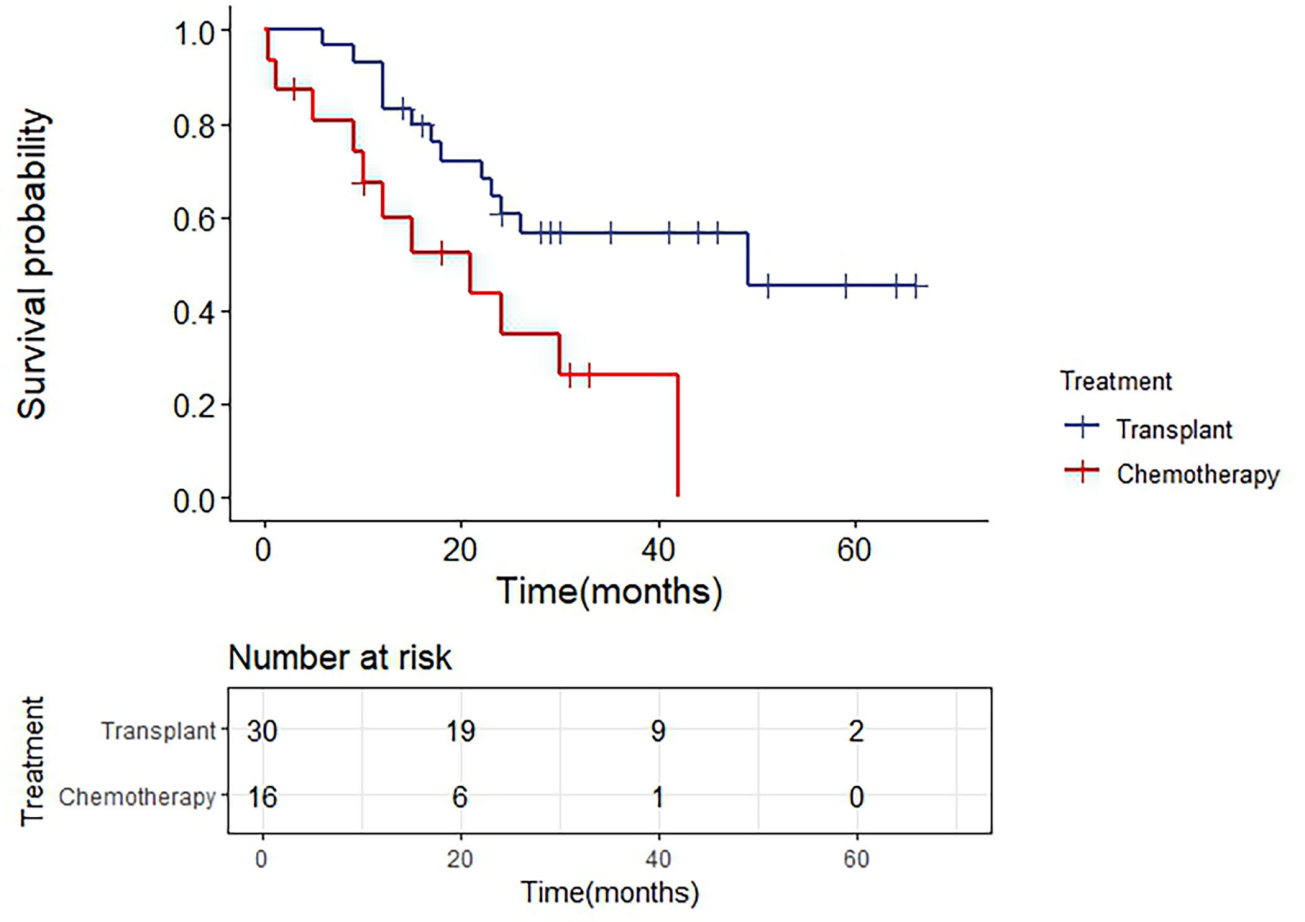
Survival analysis of SET-CAN/NUP214 fusion gene positive T-ALL patients.

In this review, we screened 46 effective cases from 49 T-ALL patients in **Table 3** (excluding No. 32, No. 34 and No. 56), 30 patients received transplantation, of which 13 died with a median survival of 49 months, 16 patients received chemotherapy, of which 11 died with a median survival of 20 months. The difference between the two groups was tested to be statistically significant (P=0.012). We listed the Kaplan-Meier survival curves of the patients in **Figure 3**, and it is clear that for T-ALL patients, transplantation can significantly improve the survival status and prolong the overall survival.

CAR-T may play a role in acute leukemia patients with positive fusion gene. The expression frequency of CD7 in previous cases is close to 100%. Research shows that CD7 may play a role in promoting chemoresistance and accelerating disease progression in leukemia (63, 64). Gomes-silva et al. (65) demonstrated that CAR-T targeting CD7 can delay disease progression and prolong patient survival in the mouse model. In the MPAL case reported by Li MY et al. (27) (no. 3), they performed two times of CAR-T cell infusion treatment on the patients who had relapsed after HSCT, which significantly improved the patient’s condition. By the end of follow-up, the patients had survived for more than 42 months. It suggests the application prospect of CAR-T technology in the treatment of fusion gene positive leukemia, which is worthy of further exploration and research.

In this review, 12 patients(12/70) with fusion gene positive AML were included, and the number was second only to T-ALL. Although the chemotherapy regimen of 12 patients was not the same, they all achieved complete remission(CR, 12/12, 100%), all patients received HSCT. Finally, 9 patients survived, 3 patients died and 4 patients relapsed. Chen SM et al. (28) showed that the survival data of *SET-CAN/NUP214* fusion gene positive AML patients were similar to those of fusion gene negative patients.

To date, only four patients with fusion gene positive B-ALL have been reported. Similarly, their treatment process was very difficult. The two patients reported by Nowak NJ et al. (32) and Hong HZ et al. (4) were resistant to chemotherapy, and have not achieved complete remission. Unfortunately, the report didn’t mentioned the follow-up of the two patients. One of the two patients(No.20-21) reported by Chen SM et al. (28) achieved complete remission, and both patients received HSCT, but they died of graft-versus-host disease (GVHD) and relapse respectively 9 and 15 months after transplantation. Although the sample of related B-ALL cases is small, we can still speculate that the patients with *SET-CAN/NUP214* fusion gene positive B-ALL may have poor prognosis.

Chen Y et al. (5) first reported two rare cases of *SET-CAN/NUP214* fusion gene positive CML in 2022 (No.18-19), the two patients detected *BCR-ABL1* and *SET-CAN/NUP214* fusion transcripts after 7 and 2 years of treatment with tyrosine kinase inhibitor (TKI), one patient (no.18) received chemotherapy and HSCT, and still survived up to the end of follow-up (95.7 months after initial diagnosis, 6.5 months after transplantation), the other patient (No.19) gave up treatment and died 36 months after the initial diagnosis. Retrospective analysis of samples from two patients showed that *SET-CAN/NUP214* fusion transcript was present at the initial diagnosis, but not during TKI treatment. The disease progression of CML is slow and typically categorized into three phases. The chronic phase (CP) is often asymptomatic but may include mild fatigue, emaciation, and splenomegaly on physical examination. The accelerated phase (AP) is characterized by fever, progressive splenomegaly, and the appearance of additional chromosomal abnormalities. The acute transformation stage (BP) is marked by the continued deterioration of symptoms and signs. Additional chromosome abnormalities play an important role in the deterioration of CML in chronic phase (CP) and accelerated phase (AP), *SET-CAN/NUP214* fusion gene may be used as the main clone in CML to promote disease transformation, and its combination with *BCR-ABL1* accelerates disease progression. Similar to the treatment of other fusion genes in CML cases, high intensity TKI chemotherapy and HSCT may be more effective for these patients (5, 66).

*SET-CAN/NUP214* fusion gene has also been found in AUL, MS and MPAL. The incidence of AUL is relatively rare. It is considered to be the result of clone expansion and maturation stagnation of undifferentiated hematopoietic cells, and does not express myeloid or lymphoid specific antigen. MS is a limited tumor formed by the proliferation and infiltration of myeloid primitive cells or immature myeloid cells outside the marrow. It may occur in association with various myeloproliferative disorders or in isolation. The lesions are mostly located in a single site, and sometimes multifocal or multiorgan involvement is present (67, 68). In this review, a case of *SET-CAN/NUP214* fusion gene positive MS patient (No.2) was included. During the treatment, the patient also suffered from bone marrow compression and pericardial effusion. The incidence of MPAL in acute leukemia is relatively low, accounting for only 2-5% of acute leukemia cases. At present, MPAL lacks a unified treatment option, and the prognosis of patients is usually worse than AML or ALL (69). Li MY et al. (27) treated a 29-year-old *SET-CAN/NUP214* fusion gene positive MPAL patient identified by them (**Table 3**, No.3) with induction and consolidation therapy leading to CR and transplanted the patient, but the patient relapsed six months later, followed by a lymphocyte consumption program based on fludarabine (30 mg/m^2^, 1-3days) and cyclophosphamide (300 mg/m^2^, 1-3days) and CAR-T cell therapy. The patient ultimately survived greater than 42 months. Chen SM et al. (28) used the treatment regimen CODLP or VPIA (vincristine+prednisone+daunorubicin+cytarabine) and transplantation for two patients(**Table 3**, No.4-5) with positive *SET-CAN/NUP214* fusion gene positive MPAL who were 22 years old and 34 years old. Both patients ultimately survived to the end of the follow-up period(survival>42 months and>24 months).

### 5.4 Prognosis prediction based on the expression level of SET-CAN/NUP214 fusion gene

Among the 70 patients counted in **Table 3**, 28 patients relapsed and 30 patients died. Relapse and death are common clinical outcomes in *SET-CAN/NUP214* fusion gene positive leukemia. We need to monitor the prognosis of patients with some indicators and detection methods, so as to better evaluate the condition of patients and timely intervene.

Current research shows that the detection of *SET-CAN/NUP214* fusion gene may be a minor residual disease (MRD) with early recurrence, or an early indicator of poor prognosis (24). Chen SM et al. carried out a long-term continuous monitoring of *SET-CAN/NUP214* gene transcript level in 24 patients, and learned that the expression level of fusion gene was lower than 0.001% continuously, which was a sign of good prognosis. The median time of morphological relapse in patients with expression level higher than 0.001% was only 5 months. Gao MG et al. (41) studied the prognostic significance of fusion gene expression level before and after allogeneic hematopoietic stem cell transplantation for patients. The expression level of fusion gene after transplantation is higher than 0.02%, which is an effective indicator of patients’ relapse. Monitoring the expression level of *SET-CAN/NUP214* fusion gene through RQ-PCR is more sensitive than flow cytometry (FCM), its sensitivity for detection of various genetic abnormalities and mutation types can reach 10^-5^, whereas the sensitivity of FCM is usually at 10^-4^ (70). 4 of the 5 patients with relapse after transplantation have *SET-CAN/NUP214+* before relapse, and their FCM detection results are negative. Previous studies also emphasized the significance of MRD monitoring in transplantation. Positive MRD before transplantation may indicate poor prognosis after transplantation (70, 71).

## 6 Conclusion

In summary, *SET-CAN/NUP214* fusion gene is relatively rare in leukemia and mainly occurs in adult male T-ALL patients. It has also been reported in AUL, MS, MPAL, AML, CML and B-ALL. Patients are generally resistant to chemotherapy, and the prognosis in different diseases may be different. The clinical symptoms of positive and negative fusion gene patients are relatively similar, and the common immunephenotypes are CD7, cCD3, CD34, CD33 and CD13. The karyotypes may be normal or complex, the concomitant molecular events can become the influencing factors of disease progression and prognosis. HSCT can significantly improve the survival rate of patients, CAR-T is also a potential treatment method. RQ-PCR is an effective monitoring method, and the monitoring of fusion gene may be more sensitive than FCM. Prognosis prediction and recurrence intervention based on the expression level of *SET-CAN/NUP214* fusion gene can improve the treatment effect. Further research is needed to evaluate the role of *SET-CAN/NUP214* fusion gene in leukemia.

## Author contributions

JS: Data curation, Investigation, Methodology, Software, Visualization, Writing – original draft. HL: Conceptualization, Funding acquisition, Project administration, Supervision, Writing – review & editing. SF: Project administration, Supervision, Writing – review & editing.
